# The association between migraine and gut microbiota: a systematic review

**DOI:** 10.1007/s13760-025-02779-y

**Published:** 2025-04-03

**Authors:** Alon Gorenshtein, Kamel Shihada, Liron Leibovitch, Tom Liba, Avner Goren

**Affiliations:** 1 Azrieli Faculty of Medicine, Bar-Ilan University, Safed, 1311502 Israel; 2 Rambam Medical Center, Haifa, Israel; 3 Maccabi Healthcare Services, Tel Aviv, Israel

**Keywords:** Migraine, Microbiome, Female, Gut-brain axis

## Abstract

**Introduction:**

Recent studies suggest a link between gut microbiota and neurological diseases, implicating the microbiome’s role in neurological health. However, the specific alterations in the microbiome associated with migraine remain underexplored. This study aims to systematically review the existing literature to determine whether migraine patients are associated with changes in gut microbiota composition.

**Methods:**

A systematic review was conducted in accordance with the PRISMA statement. We included original empirical studies investigating the microbiome in migraine patients. Data extracted included study design, participant demographics, microbiome differences at various taxonomic levels, and measures of microbial diversity (alpha and beta diversity). The search and selection process involved four independent reviewers who assessed abstracts and full texts to ensure eligibility. The gut microbiota was evaluated using relative abundance and diversity indices.

**Results:**

Six studies, encompassing various regions including China, Korea, and Italy, were included in the analysis. The results indicated significant differences in gut microbiota between migraine patients and controls. Key findings include a reduction in *Faecalibacterium*, a genus known for its anti-inflammatory properties, in migraine patients, including those with chronic migraine. Conversely, *Veillonella* exhibited elevated abundance compared to controls. Other taxa, such as *Prevotella* and *Parabacteroides*, showed variable associations with migraine across different studies, suggesting a dysbiotic gut environment in migraine patients.

**Conclusion:**

This review highlights that migraines are associated with specific alterations in gut microbiota, including decreased microbial diversity and changes in the abundance of key taxa. These findings suggest that gut microbiota dysbiosis may play a role in migraine pathophysiology. Further research is needed to explore the potential causal relationships and therapeutic implications, particularly targeting the microbiome in migraine management.

**Supplementary Information:**

The online version contains supplementary material available at 10.1007/s13760-025-02779-y.

## Introduction & background

Approximately 14% of the adult population suffers from migraine [1], with a higher incidence in females [2]. The International Classification of Headache Disorders, 3rd edition, defines migraine as a recurrent neurological condition characterized by moderate to severe headache attacks lasting 4 to 72 h, often accompanied by nausea and/or vomiting [3]. Gastrointestinal (GI) symptoms, including diarrhea, constipation, and dyspepsia, frequently co-occur with migraines and have been linked to GI conditions such as gastric stasis, gastroesophageal reflux, irritable bowel syndrome (IBS), and celiac disease [4]. The pathophysiology of migraine is multifaceted, involving genetic, environmental, and neurovascular factors. Recent research has highlighted the potential role of the gut-brain axis in migraine development, suggesting that gut microbiota may influence neurological health and disease states [5].

The gut-brain axis works bidirectionally between the GI and central nervous system (CNS). The GI system is believed to be able to affect the CNS. A number of the brain functions such as cognition, behavior, and even nociception are under the influence of the gut system [6]. Dysregulation of the gut-brain axis has been implicated in migraines as well as other neurological and psychiatric conditions, such as multiple sclerosis, mood and anxiety disorders, Alzheimer’s disease, and Parkinson’s disease [6]. A potential driver of this dysfunction is an imbalance in the gut microbiome [7]. Gut bacteria can impact the CNS through mechanisms involving microbiota-derived neurotransmitters, inflammatory mediators, and hormonal pathways [8]. Moreover, studies has speculated a relationship that the gut microbiome affect brain disease by causing a neuroinflammatory state [9]. Possible mechanism of this can be explained for migraine by gut-derived metabolites such as short-chain fatty acids (SCFAs) that regulate immune responses and neuronal excitability, contributing to the central sensitization observed in migraineurs [10]. Notably, migraine patients exhibit reduced microbial diversity and an overrepresentation of pro-inflammatory bacterial species, such as *Clostridium* spp., further supporting a mechanistic link between dysbiosis and heightened neuroinflammatory states [11].

Currently, no systematic review has specifically investigated the gut microbiota in migraine patients or compared bacterial profiles across different subgroups, including chronic migraine, pediatric migraine, and migraine comorbid with gastrointestinal (GI) diseases. This systematic review aims to identify and analyze the bacterial composition in these migraine subgroups in comparison to healthy controls, providing insights into how these bacteria may influence migraine pathology.

## Methods

This study used the following methodological framework, in conjunction with the extended Preferred Reporting Items for Systematic Reviews and Meta-Analyses (PRISMA) checklist for systemic reviews [12]. The study protocol was pre-registered on the International Prospective Register Reviews (PROSPERO; CRD42024514664).

### Search strategy and selection criteria

This systematic review included studies on individuals with migraine patients which had quantified the microbiota, sourced from databases including PubMed, Central, Scopus, and Web of Sciences to establish an extensive pool of helpful information regarding the microbiome of migraine patients. A search limit was not put in place due to the lack of studies in the area. We used a search strategy focused on identifying the microbiome and its association with the clinical symptoms of migraine. The following search strategy for PubMed was used: (“Migraine Disorders“[Mesh] OR “Headache Disorders“[Mesh] OR “Migraine Disorders” OR “Migraine” OR “Migraines” OR “Migraine Headache” OR “Migraine Headaches” OR “Acute Confusional Migraine” OR “Status Migrainosus” OR “Abdominal Migraine” OR “Cervical Migraine Syndrome” OR “Vestibular Migraine” OR “Ophthalmoplegic Migraine” OR “Retinal Migraine” OR “Complicated Migraine” OR “Hemiplegic Migraine” OR “Basilar-Type Migraine” OR “Migraine with Aura” OR “Migraine without Aura” OR “Chronic Migraine” OR “Menstrual Migraine”)

AND (“Microbiota“[Mesh] OR “Dysbiosis“[Mesh] OR “Gastrointestinal Microbiome“[Mesh] OR “Probiotics“[Mesh] OR “Prebiotics“[Mesh] OR “Microbiota“[Mesh] OR “Microbiota” OR “Microbiome” OR “Gut Microbiota” OR “Intestinal Microbiota” OR “Gastrointestinal Microbiome” OR “Gut Microbiome” OR “Gut Flora” OR “Intestinal Flora” OR “Gut Bacteria” OR “Intestinal Bacteria” OR “Commensal Bacteria” OR “Symbiotic Microorganisms” OR “Dysbiosis” OR “Microbial Ecology” OR “Microbial Community” OR “Microbial Diversity” OR “Microflora”).

### Inclusion and exclusion criteria

This review investigated migraine patient’s microbiome in comparison to healthy control patient microbiome. We excluded studies that reported generalized microbiome assessment without detailed quantified measurement. Additionally, non-English language studies, case reports, case series, systematic reviews, and meta-analyses were also excluded.

### Data extraction and quality assessment

Screening and data extraction was meticulously conducted by four independent reviewers (A.G., K.S, T.L. and L.L.). Each reviewer independently screened the studies for inclusion. In cases where discrepancies arose during the screening phase, they were discussed among all four reviewers to reach a consensus. If a unanimous decision could not be achieved, the issue was referred to a senior reviewer (Avner Goren) for final resolution. Only data pertaining to the patient groups were extracted. A standardized extraction tool was employed to collect relevant information, including the first author’s name, study location, publication date, sample size, number of migraine patients, number of controls, and the technical and computational methods used for processing and quantifying the microbiota. Four authors (A.G., K.S, T.L. and L.L.) independently extracted data from the full texts of the six studies that passed the initial screening. Any inconsistencies in the extracted data were discussed collectively, and if necessary, a senior reviewer (Avner Goren) was consulted to resolve persistent conflicts. During the quality assessment phase, two authors (A.G. and K.S.) independently evaluated the studies based on several criteria: the classification of migraine, the quantified measurement to assess microbiome, and the objectives related to evaluating microbiome difference between case and control. In instances where disagreements occurred between the reviewers during this phase, the senior reviewer (Avner Goren) provided the decisive judgment to resolve these issues.

### Risk of bias assessment

Risk of bias assessment was performed using the Newcastle–Ottawa Scale (NOS) to critically appraise the literature included in the systematic review using common variables. Methodological quality and risk of bias were assessed by independent reviewers according to the NOS, which is valid for use in cohort studies [13] and the adapted version for cross-sectional studies [14]. The scale consists of eight items with three quality parameters: (i) selection, (ii) comparability, and (iii) outcome. The quality of the studies (poor, fair, and good) was scored by allocating stars to each domain as follows: a poor-quality score was allocated 0 or 1 star(s) in the selection, 0 stars in comparability, and 0 or 1 star(s) in the outcome domain; a fair quality score was awarded, two stars in the selection, one or two stars in comparability, and two or three stars in outcomes. A good quality score was awarded, with three or four stars in selection, 1 or 2 in comparability, and two or three stars in outcomes.

## Results

**Fig. 1:**
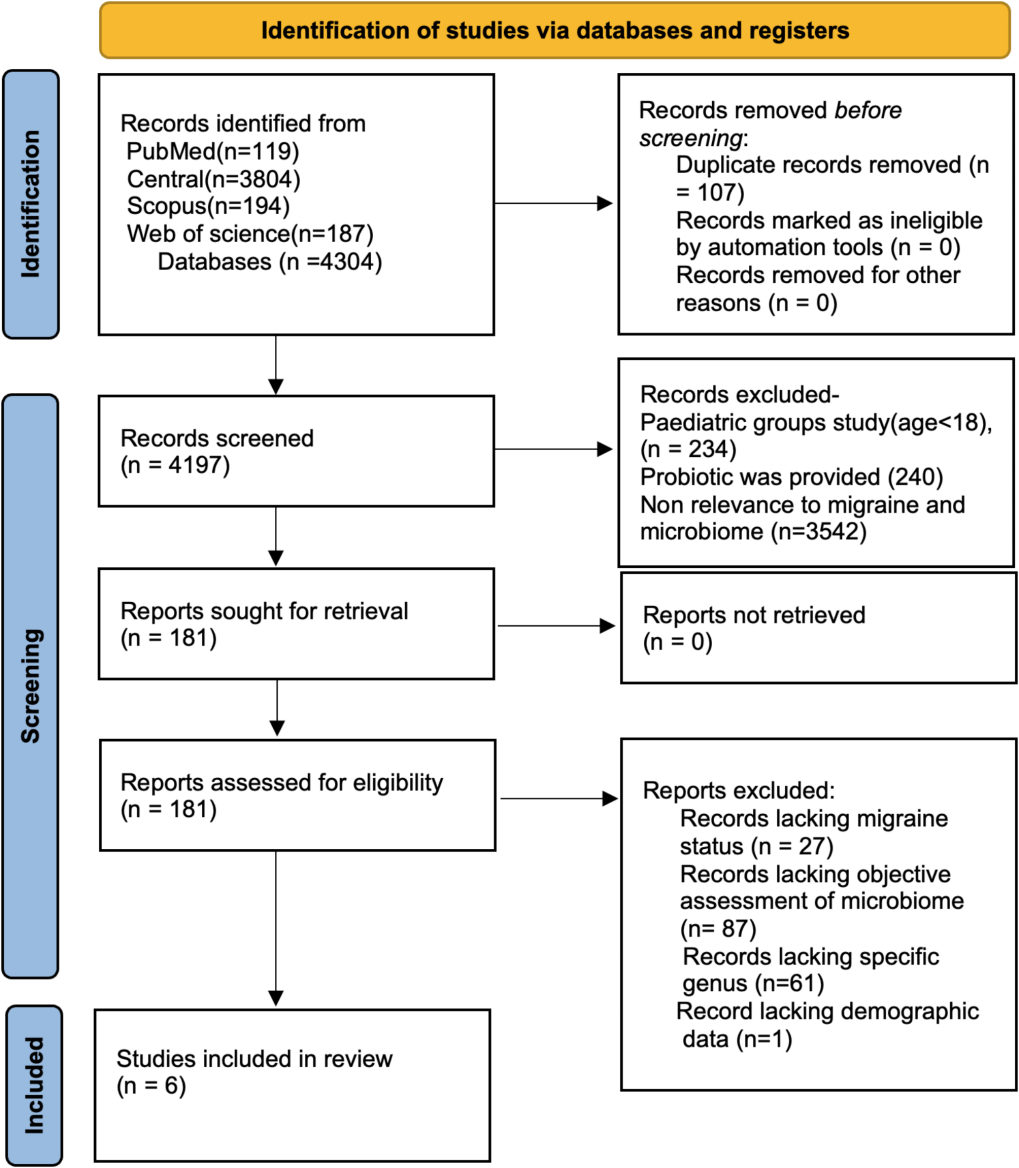
Preferred reporting items for systematic reviews and meta-analyses (PRISMA) Flow Chart

In total, 4304 articles were reviewed after the removal of duplicates, 4197 articles were screened for titles and abstracts, and 181 articles met the criteria. These studies presented data on migraine patients in relationship to the microbiome. Afterward, the full texts of these articles were evaluated following the inclusion and exclusion criteria that were issued above, and after quality assessment, a total of six studies were included in this review [15–20], Fig. 1 showcase flow diagram detailing the review process and study selection based on the PRISMA flow chart.

### Risk of bias in included studies

Table 1 summarizes our assessments of the risk of bias in the included studies. We rated all the studies with an overall low risk of bias of the observational studies as “good”.

**Table 1:** Risk of bias quality assessment

			Selection			Comparability			Exposure/Outcome			Sub total assessment		Conclusion	Total
	Study type	1	2	3	4	1a	1b	1	2	3	S	C	E		
Jiang et al.	Cross-sectional	*	No	*	**	*		*	*	*	Good	Good	Good	Good	8
Yong et al.	Cross-sectional	*	No	No	No	*	*	*	*	No	Fair	Good	Good	Good	7
Chen et al.	Case-control	*	No	No	*	*	*	*	*	No	Fair	Good	Good	Good	5
Liu et al.	Case-control	*	No	No	*	*	*	*	*	*	Fair	Good	Good	Good	8
Jieqiong Liu et al.	Case-control	*	*	No	*	*	*	*	*	No	Good	Good	Good	Good	8
Papetti et al.	Case-control	*	*	No	*	*	*	*	*	*	Good	Good	Good	Good	9

### Characteristics of the results

The sample size ranged from 29 to 196. The earliest publication date was 21/06/2021 while the latest was 09/10/2024. This indicates that investigations into the relationship between the microbiome and migraine are relatively nascent, covering a very recent and brief temporal span. Three countries were included in the review: China, Italy, and Korea. The majority of the studies (*n* = 4) originated from China. All six studies used a case-control design for a total of 314 migraine cases and 279 controls. Five studies collected stool samples from patients while one collected saliva [15]. Across the six studies, there was female predominant (80.06%), while one study lacked demographic data for the control group [19]. Migraine cases had slightly higher females in comparison to control (82% vs. 79%). The mean ages of the case and control groups across studies were 34.33 ± 7.78 and 37.95 ± 6.60, respectively. Diet assessment was mentioned in only two studies. In one, it was noted that all participants shared similar dietary habits and had lived in the same city for at least three months prior to sample collection [12], while in the second study the authors stated that no significant difference between the migraine-irritable bowel syndrome (M-IBS) group and IBS group in eating habits [19]. Six of the studies explored episodic migraine, one of which also investigated chronic migraine. In that study, 47 patients had chronic migraine, and 42 had episodic migraine [16] (Table 2).

**Table 2:** The migraine gut microbiota: summary of the included studies

References	Country	Published time	Study type	No. of patients with migraine	Mean age migraine patients	Key findings
Jiang et al.	China	21/06/2021	Cross-sectional, case-control	26	39.5	Significant difference in the composition of oral microbiota between migraine patients and healthy individuals, with 23 genera were found differentially abundant between migraine and control groups
Yong et al.	Korea	12/01/2023	Cross-sectional, case-control	87	39.6 ± 11.4- episodic migraine, 40.8 ± 12.5- chronic migraine	The composition of microbiota differed significantly between the 3 groups: episodic migraine, chronic migraine and control groups
Chen et al.	China	29/01/2020	Case-control	54	61	Significant difference in species diversity and altered microbiota between migraine and controls and difference in metabolic functions in the gut microbiota of migraine patients
Liu et al.	China	05/01/2024	Case-control	33	7.48 ± 1.95	Altered microbiota at multiple levels of taxa were identified in pediatric migraine children, especially the genera that regulate tryptophan metabolism, in addition significant decrease in plasma kynurenic acid levels, increase in serotonin and quinolinic acid in migraine patients
Jieqiong Liu et al.	China	16/11/2022	Case-control	16 with M-IBS	39.69 ± 11.57	Significant increase of bacterial richness and gut dysbiosis in the pediatric migraine patients, thirty-seven metabolic pathways were increased in the migraine group, which includes changes in tryptophan and phenylalanine metabolism
Papetti et al.	Italy	09/10/2024	Case-control	98	12.5 ± 2.87	The composition of microbiota differed between migraine patients with IBS and patients with IBS only, while the richness and diversity of gut microbiota in migraine patients with IBS showed no significant difference

### Technical, computational methods used to process and quantify the microbiota

Of the six studies, four specified methods for sample collection, focusing on different regions of the 16 S rRNA gene sequenced with Illumina MiSeq and one with Illumina HiSeq. OTUs were generated using QIIME for standardized microbial classification. In contrast, Chen et al. applied shotgun metagenomics, bypassing OTU clustering by capturing comprehensive genomic data, while Liu et al., utilizing preprocessed data from the GMrepo database, conducted principal coordinates analysis (PCoA) to evaluate community structure.

### Gut microbiota diversity

All studies assessed gut microbiota diversity by using alpha- and beta-diversity metrics. Alpha diversity refers to the richness, evenness, and overall variety of microbial taxa within an individual sample, with common indices including Shannon, Simpson, and Chao1. In contrast, beta diversity evaluates the differences in microbial composition between samples or groups, often using Bray-Curtis, weighted UniFrac, and unweighted UniFrac metrics.

Regarding alpha diversity, the findings were inconsistent across studies. Some studies reported significantly higher diversity in migraine patients, while others observed reduced diversity or no significant differences at all. For instance, Jiang et al. found that migraine patients exhibited significantly greater oral microbiota diversity compared to control subjects, as evidenced by higher scores on the Shannon (*p* = 0.0016), Simpson (*p* = 0.0018), Chao1 (*p* = 0.02), observed species (*p* = 0.0057), and Phylogenetic Diversity (PD) whole tree (*p* = 0.045) indices. Similarly, Papetti et al. observed a significant increase in bacterial richness among pediatric migraineurs, with substantial differences in the Shannon (*p* < 0.001), Simpson (*p* = 0.0006), and Chao1 (*p* = 0.001) indices.

In contrast, other studies reported decreased alpha diversity in migraine patients. Chen et al. found a significant reduction in diversity at both the genus (*p* = 0.036) and species (*p* = 0.048) levels, based on the Shannon index. Liu et al. also found significantly lower gut microbiota richness and evenness in migraine patients, as indicated by the Shannon and Simpson indices (all *p* < 0.001).

A few studies, however, found no significant differences in alpha diversity between migraine patients and controls. Jieqiong Liu et al. reported no notable differences in alpha diversity between patients with M-IBS and those with IBS alone. Similarly, Yong et al., who assessed episodic and chronic migraine separately, found no statistically significant differences in alpha diversity across these groups.

When it comes to beta diversity, most studies observed significant differences in the microbial composition between migraine patients and controls, though a one have reported no such differences. Jiang et al. noted significant differences using both weighted UniFrac (*p* = 0.001, R²=0.132) and unweighted UniFrac (*p* = 0.002, R²=0.065). Likewise, Chen et al. found significant compositional differences using the Bray-Curtis dissimilarity metric (PERMANOVA, *p* = 0.006, R²=0.014). Papetti et al. similarly observed significant differences in microbial composition using Bray-Curtis (*p* = 0.001).

In line with these findings, Jieqiong Liu et al. and Liu et al. also reported significant beta diversity differences using Bray-Curtis principal coordinate analysis (*p* = 0.041 and *p* = 0.001, respectively) and partial least squares discrimination analysis (*p* < 0.001). These results suggest that the microbiota composition of migraine patients is distinct from that of controls.

In contrast, Yong et al. found no significant differences in beta diversity, with all metrics—weighted UniFrac (*p* = 0.176), unweighted UniFrac (*p* = 0.132), and Bray-Curtis dissimilarity index (*p* = 0.22), showing no statistically significant variations between episodic and chronic migraine groups. These diverse methodological approaches across the studies emphasize the range of analytical techniques employed to characterize microbiota in relation to migraine (Table 3) [15–20].

**Table 3:** Technical and computational methods used to process and quantify the microbiota

References	Microbiota Quantifying Instrument, 16 S rRNA Region	Method Generating OTUs	Sample type	Beta Diversity metric findings between migraine and control	Alpha diversity metrices findings between migraine and control	Alpha diversity metrices finding between chronic migraine and control
Jiang et al.	Illumina MiSeq,	QIIME	Oral	Weighted (*p* = **0.001**, R^2 = 0.132); Unweighted Unifrac (*p* = **0.002**, R^2 = 0.065)	Shannon (*p* = **0.0016**), Simpson (*p* = **0.0018**), Chao1 (*p* = **0.02**), Observed species (*p* = **0.0057**), Faith’s Phylogenetic Diversity (PD) whole tree (*p* = **0.045**)	NA
Yong et al.	Illumina MiSeq	EzBioCloud database	Fecal	Weighted (*p* = 0.176); Unweighted; Unifrac (p–=0.132); Bray–Curtis dissimilarity index (*p* = 0.22)	Shannon (*p* = 0.28), Chao1 (*p* = 0.55, Simpson(*p* = 0.23)	Shannon (*p* = 0.085), Chao1 (*p* = 0.67, Simpson(*p* = 0.12)
Chen et al.	Shotgun metagenomics	Shotgun metagenomics	Fecal	Bray–Curtis -PERMANOVA (*p* = **0.006**, R^2 = 0.014)	Genus Shannon (*p* =** 0.036**), Species Shannon (**0.048**)	NA
Liu et al.	NA (GMrepo database)	GMrepo database	Fecal	Bray–Curtis - principal coordinates analysis (*p* = **0.001**, R^2 = 0.076)	Shannon index and Simpson index (all *p* < **0.001**)	NA
Jieqiong Liu et al.	Illumina HiSeq 2500	QIIME	Fecal	Bray–Curtis -principal coordinate analysis (*p* = **0.041**); partial least squares discrimination analysis (*p* < **0.001**)	chao1 (*p* = 0.487); Observes species (*p* = 0.661); PD whole tree p= (0.358); Shannon (*p* = 0.546); Simpson (*p* = 0.408)	NA
Papetti et al.	Illumina MiSeq	QIIME	Fecal	Bray–Curtis -PERMANOVA (*p* = **0.001**)	Shannon index: (*p* < **0.001**); Simpson index (*p* = **0.0006**); Chao 1 (*p* = **0.001**)	NA

Bold values are statistical significance at *p* < 0.05

### Taxa-level findings

Taxa-level analysis refers to examining microbiome differences at various taxonomic ranks (e.g., phylum, family, genus, species). This analysis revealed notable microbiome differences between migraine patients and controls across multiple taxonomic levels, as shown in Table 3, suggesting a potential role of these genera in migraine pathophysiology.

At the genus level, *Prevotella* displayed a complex pattern, with elevated levels in episodic migraine cases compared to chronic cases and controls, indicating its potential as a microbial marker for episodic versus chronic migraine subtypes.

Distinct microbial shifts were also observed in special groups, including pediatric migraine patients and those with comorbid IBS. In pediatric cases, Liu et al. identified taxa involved in tryptophan metabolism, with significant reductions in plasma kynurenic acid levels and increases in serotonin and quinolinic acid, suggesting a distinct metabolic and neuroinflammatory profile in children with migraine.

Interestingly, *Porphyromonas* and *Dialister*, displayed variability in abundance showing higher levels in elderly migraineurs but reduced levels in pediatric cases. These differences could reflect age-related differences in the gut microbiome, or varying immune responses across different life stages.

Additional genera such as *Veillonella* demonstrated consistently elevated abundances in migraine cases across studies. Known for its involvement in lactate metabolism, *Veillonella*’s association with migraine suggests a link to metabolic processes that could influence migraine pathogenesis.

Beneficial genera like *Faecalibacterium* and *Roseburia*, known for their anti-inflammatory properties and roles in gut homeostasis, were significantly reduced in chronic migraine cases. While *Roseburia* was generally reduced, one study assessing pediatric migraine patients reported elevated levels. In contrast, *Faecalibacterium* consistently showed lower levels across all studies, with one study specifically reporting a reduction in *Faecalibacterium Prausnitzii*. Such alterations in gut microbiota may promote pro-inflammatory pathways, potentially contributing to migraine pathogenesis.

Overall, these findings suggest that migraine is associated with distinct shifts in the gut microbiome, characterized by elevated potentially pathogenic genera such as *Veillonella* and reductions in health-promoting bacteria like *Faecalibacterium* and *Roseburia* (Table 4) [15–20].

**Table 4:** Key findings from clinically relevant taxa-level relative abundances: migraine cases versus controls

	Jiang et al.	Yong et al.*	Papetti et al.	Chen et al.	Jieqiong Liu et al.	Liu et al.
Genus						
Dialister	↑	NR	NR	NR	NR	↓
Veillonella	↑	NR	NR	NR	NR	↑
Porphyromonas	↑	NR	NR	NR	NR	↓
Prevotella	↑	↑ in episodic migraine vs. chronic migraine	NR	NR	NR	↓
Faecalibacterium	NR	↓ in chronic migraine vs. control	NR	↓	NR	↓
Eggerthella	NR	↑ in chronic vs. Control	↓	NR	NR	↑
coprococcus	NR	↑ in episodic migraine vs. chronic migraine	NR	NR	NR	↓
Roseburia	NR	↓ in episodic migraine vs. control ↓ in chronic migraine vs. control	↑	NR	NR	NR
Collinsella	NR	NR	↓	NR	↓	NR
Parabacteroides	NR	NR	↓	NR	↑	↑
Species						
Prevotella copri	NR	NR	NR	↓	NR	NR
Phylum						
Bacteroidetes	NR	NR	NR	NR	NR	↑

*Yong et al. explored both episodic and chronic migraine microbiome

## Discussion

Our systematic review identified distinct microbial signatures associated with migraine, offering insights into its pathophysiology and potential microbiome-based therapeutic targets. From a clinician’s perspective, these findings can point out how future interventions aim at modifying gut microbiota (e.g., dietary changes, probiotics, and prebiotics) might complement standard pharmacological approaches to migraine. While alpha diversity did not significantly differ between migraine patients and controls in two studies [16, 20]. Specific subgroups, including pediatric, elderly, exhibited higher diversity [15, 19], suggesting the presence of dysbiosis in these populations. In addition, increased diversity within the oral microbiota was observed, indicating that alterations in the oral microbial composition may be significant for individuals with migraines [15]. Importantly, epigenetic factors may modulate how this dysbiosis translates into neuroinflammatory processes, as environmental exposures (e.g., diet, stress) can induce heritable changes in gene expression that amplify migraine-related pathways [21]. Consequently, understanding the interplay between the gut microbiome, epigenetic mechanisms, and conventional therapies could pave the way for more personalized management strategies in migraine.

The strong association between migraine and neurological conditions may be attributed to impairments in the brain-gut axis mediated by an inflammatory state. The proposed mechanisms involve a range of processes, including metabolite production, modulation of gut barrier function, immune regulation, neurotransmitter synthesis, and signaling through the vagus nerve [22–24].In our review bacteria associate with nitrate homeostasis such as *Prevotella* and *Veillonella* [25], were identified in high abundance in studies by Jiang et al., Yong et al., and Liu et al. (*Prevotella* was found in lower amounts in Liu et al.‘s study, while *Veillonella* was not reported in Yong et al.‘s). These bacteria have been correlated with increased plasma nitrite levels in response to nitrate supplementation, suggesting their role in nitric oxide (NO) homeostasis. Taking into consideration that NO is a classic migraine trigger [26], and involves in nociceptive transmission and increased NO stress in migraine patients [27], we would propose a potential link between migraine and NO-associated bacteria.

A key element of this neuroinflammatory pathway involves nitric oxide (NO) metabolism, long implicated in migraine pathogenesis through its vasodilatory properties and capacity to sensitize trigeminal neurons [28]. Several bacterial genera, including *Prevotella* and *Veillonella*, influence nitrate-to-nitrite conversion, affecting systemic NO levels [25]. Experimental data further indicate that recurrent NO exposure can disrupt gut microbial diversity by reducing beneficial anaerobes (e.g., *Faecalibacterium prausnitzii*) and increasing potentially pathogenic taxa, thereby fueling an inflammatory milieu [29]. Our systematic review points to both NO-producing and NO-modulating bacteria such as *Prevotella* and *Veillonella*, as particularly relevant in migraine populations. Collectively, these findings highlight a potential vicious cycle, where abnormal NO metabolism promotes dysbiosis-driven inflammation, further exacerbating migraine-related neuroinflammation [30].

Migraine patients was also associated with lack of many beneficial bacteria, such as *Faecalibacterium* [31], a key butyrate-producing taxon widely recognized for its robust anti-inflammatory effects [32]. Moreover there are studies reporting lower levels of *Faecalibacterium* in neuropsychiatric diseases such as depression and anxiety, conditions often associated with unhealthy diets [33, 34]. By increasing IL-10 secretion and modulating both innate and adaptive immunity, *Faecalibacterium prausnitzii* could help attenuate neuroinflammation relevant to migraine pathophysiology [35]. In theory, patients may find *Faecalibacterium prausnitzii’s* butyrate production beneficial, this can be explained by prior study that showed treatment with SCFAs like butyrate was associated with lessened migraine symptoms in a mouse model [36]. Due to their favorable effect, a reduction in these bacteria may disrupt the brain-gut axis by impairing the production of energy for colonocytes and anti-inflammatory metabolites critical for intestinal health [37]. Moreover, butyrate is a principal metabolite that can cross the blood-brain barrier and potentially dampen microglial activation and trigeminal nociceptive signaling, thereby offering a mechanistic link to migraine severity or frequency [37]. Although direct evidence tying *Faecalibacterium* abundance to improved migraine outcomes remains scarce, these multi-level anti-inflammatories and neuromodulatory effects suggest a promising avenue for microbiome-based therapies [10]. Consequently, strategies aimed at elevating *Faecalibacterium* whether through probiotic, prebiotic, or dietary interventions, may help restore gut-brain homeostasis and provide an adjunctive approach for mitigating migraine burden [10, 38]. One possible mechanism is the modulation of neurotransmitter systems central to migraine pathophysiology [39]. Certain taxa can synthesize or degrade neurotransmitters like serotonin, dopamine, and gamma-aminobutyric acid (GABA), influencing neuronal excitability and pain perception [39, 40].

Another gut microbiome identified in our review with inflammatory effects is *Parabacteroides*, which has been associated with migraine patients. *Parabacteroides* belongs to the phylum *Bacteroidetes*, a group of Gram-negative anaerobic bacteria that commonly colonize the human digestive system [41]. This bacterium contributes to inflammation through mechanisms involving lipopolysaccharides (LPS) and the metabolic end-product succinate [42]. In addition to these findings, a recent meta-analysis examined the association between *Helicobacter pylori (H. pylori)* infection and migraine, identifying a significant correlation between the two conditions. Bawand et al. reported that *H. pylori* infection was associated with an increased risk of migraine, with an overall odds ratio (OR) of 2.80 (95% CI = 1.75–4.48), suggesting that infected individuals are nearly three times more likely to suffer from migraines compared to uninfected individuals [43]. A report indicated that chronic *H. pylori* infection might contribute to systemic inflammation and neuroinflammatory responses implicated in migraine pathophysiology [44]. Additionally, a clinical trial suggested that eradication therapy against *H. pylori* may reduce migraine frequency and severity in selected patients [45, 46], highlighting a potential therapeutic avenue.

Importantly, headache medications may influence gut microbiota composition, potentially contributing to dysbiosis. A study observed aspirin use is associated with changes in *Prevotella* and *Bacteroides* species, while Celecoxib and ibuprofen use linked to enrichment of Acidaminococcaceae and Enterobacteriaceae, suggesting that these agents can alter gut microbial communities [47]. 

Our findings highlight the pivotal role of inflammation in the brain-gut axis of migraine patients, particularly through the link between gut bacteria and an inflammatory state. Proinflammatory cytokines, including IL-1β, IL-6, IL-8, and TNF-α, are elevated during migraine attacks, sensitizing nociceptive pathways such as the trigeminal system [47–49, 48–50]. Gut microbiota exerts its influence through indirect signaling mechanisms involving microbiota-derived neurotransmitters, inflammatory molecules, and SCFAs [10]. These components play a critical role in maintaining gut barrier integrity and modulating systemic immunity. Dysbiosis, often induced by stress or dietary changes, disrupts these processes, leading to increased gut permeability, LPS leakage, and cytokine-driven inflammation. This cascade further amplifies pain pathways associated with migraines [51, 52]. Evidence from germ-free murine models showcase the protective role of gut microbiota in mitigating hypernociception induced by inflammation. These models exhibit reduced nociceptive responses compared to conventional counterparts, highlighting the influence of gut microbiota in regulating pain pathways [53]. Additionally, dietary interventions, including increased fiber intake and probiotic supplementation, have been shown to restore butyrate-producing bacteria, thereby mitigating inflammation [54, 55]. These findings, along with our observations, emphasize the critical role of gut microbiota in regulating inflammation and influencing migraine pathophysiology, presenting promising opportunities for the development of targeted therapeutic interventions.

Several limitations warrant consideration. First, the studies included in this review exhibit substantial heterogeneity in diagnostic criteria, population characteristics, and microbiome assessment methods (e.g., 16 S rRNA vs. whole-genome sequencing), complicating cross-comparison of results. Second, most studies lacked rigorous dietary control, yet diet is a major determinant of microbial composition and could confound associations between specific taxa and migraine. Third, the relatively small sample sizes and diverse geographical settings across the studies introduce potential selection bias and limit generalizability. Future multicenter trials with standardized methodologies, carefully controlled diets, and larger cohorts are necessary to validate the robustness of current observations.

In conclusion, our systematic review identifies potential gut microbiomes compositions in migraine and the mechanistic pathways by which specific bacterial taxa may contribute to migraine pathophysiology. While overt dysbiosis may not uniformly characterize adult migraine cohorts, our synthesis of current evidence proposes that shifts in key taxa can drive a pro-inflammatory response. Future research should apply longitudinal designs and mechanistic analyses to explore precise causal pathways and refine microbiome-based strategies for migraine prevention and treatment.

## Electronic supplementary material

Below is the link to the electronic supplementary material.

Supplementary Material 1

## Data Availability

No datasets were generated or analysed during the current study.
