# Supplementary material for: The association between migraine and gut microbiota: a systematic review
Source: Acta Neurol Belg. 2025 Apr 3;125(4):977–87. doi: 10.1007/s13760-025-02779-y (PMC12391207; doi:10.1007/s13760-025-02779-y)
Supplement: Supplementary file 1 — Supplementary Material 1 [file 13760_2025_2779_MOESM1_ESM.docx]

supplementary Table 1: Key findings from taxa-level relative abundances: migraine cases versus controls

|  | Jiang et al. | Yong et al.* | Papetti et al. | Chen et al. | Jieqiong Liu et al. | Liu et al. |
| --- | --- | --- | --- | --- | --- | --- |
| **Genus** |  |  |  |  |  |  |
| Treponema | ↑ | NR | NR | NR | NR | NR |
| Fretibacterium | ↑ | NR | NR | NR | NR | NR |
| Dialister | ↑ | NR | NR | NR | NR | ↓ |
| SR1 genera incertae sedis | ↑ | NR | NR | NR | NR | NR |
| Alloprevotella | ↑ | NR | NR | NR | NR | NR |
| Kingella | ↑ | NR | NR | NR | NR | NR |
| Megasphaera | ↑ | NR | NR | NR | NR | NR |
| Mycoplasma | ↑ | NR | NR | NR | NR | NR |
| Aggregatibacter | ↑ | NR | NR | NR | NR | NR |
| Campylobacter | ↑ | NR | NR | NR | NR | NR |
| Capnocytophaga | ↑ | NR | NR | NR | NR | NR |
| Saccharibacteria genera incertae sedis | ↑ | NR | NR | NR | NR | NR |
| Veillonella | ↑ | NR | NR | NR | NR | ↑ |
| Porphyromonas | ↑ | NR | NR | NR | NR | ↓ |
| Prevotella | ↑ | ↑ in episodic migraine vs chronic migraine | NR | NR | NR | ↓ |
| Rothia | ↓ | NR | NR | NR | NR | NR |
| Turicibacter | ↓ | NR | NR | NR | NR | NR |
| Granulicatella | ↓ | NR | NR | NR | NR | NR |
| Micrococcus | ↓ | NR | NR | NR | NR | NR |
| Clostridium sensu stricto | ↓ | NR | NR | NR | NR | NR |
| Lautropia | ↓ | NR | NR | NR | NR | NR |
| Methanobrevibacter | ↓ | NR | NR | NR | NR | NR |
| Lachnoanaerobaculum | ↓ | NR | NR | NR | NR | NR |
| Faecalibacterium | NR | ↓ in chronic migraine vs control | NR | ↓ | NR | ↓ |
| Bacteroides | NR | NR | NR | NR | NR | ↑ |
| parabacteroides | NR | NR | ↓ | NR | ↑ | ↑ |
| Lachnoclostridium | NR | NR | NR | NR | NR | ↑ |
| Sutterella | NR | NR | NR | NR | NR | ↑ |
| Parasutterella | NR | NR | NR | NR | NR | ↑ |
| Anaerofilum | NR | NR | NR | NR | NR | ↑ |
| Coprobacillus | NR | NR | NR | NR |  | ↑ |
| Eggerthella | NR | ↑ in chronic vs Control | ↓ | NR | NR | ↑ |
| bifidobacterium | NR | NR | NR | NR | NR | ↓ |
| rumincoccus | NR | NR | NR | NR | NR | ↓ |
| ruminiclostridum | NR | NR | NR | NR | NR | ↓ |
| corynebacterium | NR | NR | NR | NR | NR | ↓ |
| streptococcus | NR | NR | NR | NR | NR | ↓ |
| sporobacter | NR | NR | NR | NR | NR | ↓ |
| coprococcus | NR | ↑ in episodic migraine vs chronic migraine | NR | NR | NR | ↓ |
| Lactobacillus | NR | NR | NR | NR | NR | ↓ |
| Bilophila | NR | NR | NR | NR | NR | ↓ |
| Desulfovibrio | NR | NR | NR | NR | NR | ↓ |
| Tyzzerella | NR | NR | NR | NR | NR | ↓ |
| Olsenella | NR | ↑ in Episodic vs Control  ↑ episodic vs chronic migraine | NR | NR | NR | NR |
| Hungatella | NR | ↑ in chronic vs Control | NR | NR | NR | NR |
| Clostridium_g6 | NR | ↑ in chronic vs Control | NR | NR | NR | NR |
| Longicatena | NR | ↑ in chronic vs Control | NR | NR | NR | NR |
| PAC001212_g | NR | ↑ in Chronic migraine vs Episodic migraine | NR | NR | NR | NR |
| Holdemanella | NR | ↑ in episodic migraine vs chronic migraine  ↓ in chronic migraine vs control | NR | NR | NR | NR |
| Adlercreutzia | NR | ↑ in episodic migraine vs chronic migraine | NR | NR | NR | NR |
| Lachnospiraceae_uc | NR | ↓ in episodic migraine vs control | NR | NR | NR | NR |
| Eubacterium_g21 | NR | ↓ in episodic migraine vs control | NR | NR | NR | NR |
| Eubacterium_g20 | NR | ↓ in episodic migraine vs control | NR | NR | NR | NR |
| Roseburia | NR | ↓ in episodic migraine vs control  ↓ in chronic migraine vs control | ↑ | NR | NR | NR |
| Agathobacter | NR | ↓ in chronic migraine vs control | NR | NR | NR | NR |
| Fusicatenibacter | NR | ↓ in episodic migraine vs control | NR | NR | NR | NR |
| PAC000195_g | NR | ↓ in episodic migraine vs control  ↓ in chronic migraine vs control | NR | NR | NR | NR |
| Catenibacterium | NR | ↓ in episodic migraine vs control  ↓ in chronic migraine vs control | NR | NR | NR | NR |
| Lachnospira | NR | ↓ in chronic migraine vs control | NR | NR | NR | NR |
| Dorea | NR | ↓ in chronic migraine vs control | NR | NR | NR | NR |
| Frisingicoccus | NR | ↓ in chronic migraine vs control | NR | NR | NR | NR |
| Eubacterium_g4 | NR | ↓ in chronic migraine vs control | NR | NR | NR | NR |
| Agathobacter | NR | ↓ in chronic migraine vs control | NR | NR | NR | NR |
| PAC001137_g | NR | ↓ in chronic migraine vs control | NR | NR | NR | NR |
| PAC000692_g | NR | ↓ in chronic migraine vs control | NR | NR | NR | NR |
| PAC001134_g | NR | ↓ in chronic migraine vs control | NR | NR | NR | NR |
| Paraprevotella | NR | NR | NR | NR | ↓ | NR |
| Lachnospiraceae_UCG-010 | NR | NR | NR | NR | ↓ | NR |
| Lactococcus | NR | NR | NR | NR | ↓ | NR |
| Collinsella | NR | NR | ↓ | NR | ↓ | NR |
| Comamonas | NR | NR | NR | NR | ↓ | NR |
| Gemmiger | NR | NR | ↑ | NR | NR | NR |
| Phocaeicola | NR | NR | ↑ | NR | NR | NR |
| Escherichia | NR | NR | ↑ | NR | NR | NR |
| ER4 | NR | NR | ↑ | NR | NR | NR |
| Acetatifactor | NR | NR | ↑ | NR | NR | NR |
| Alistipes_A_871400 | NR | NR | ↑ | NR | NR | NR |
| Dorea_A | NR | NR | ↑ | NR | NR | NR |
| Rombustia | NR | NR | ↑ | NR | NR | NR |
| Alistipes_A_871404 | NR | NR | ↓ | NR | NR | NR |
| Clostridium | NR | NR | ↓ | NR | NR | NR |
| Erysipelatoclostridium | NR | NR | ↓ | NR | NR | NR |
| Akkermansia | NR | NR | ↓ | NR | NR | NR |
| Faecalibacillus | NR | NR | ↓ | NR | NR | NR |
| **Species** |  |  |  |  |  |  |
|  |  |  |  |  |  |  |
| Eggerthella lenta | NR | NR | NR | ↑ | NR | NR |
| Clostridium asparagiforme | NR | NR | NR | ↑ | NR | NR |
| Clostridium clostridioforme | NR | NR | NR | ↑ | NR | NR |
| Clostridium bolteae | NR | NR | NR | ↑ | NR | NR |
| B. hydrogenotrophica | NR | NR | NR | ↑ | NR | NR |
| Clostridium citroniae | NR | NR | NR | ↑ | NR | NR |
| Clostridium hathewayi | NR | NR | NR | ↑ | NR | NR |
| Clostridium ramosum | NR | NR | NR | ↑ | NR | NR |
| Clostridium spiroforme | NR | NR | NR | ↑ | NR | NR |
| Clostridium symbiosum | NR | NR | NR | ↑ | NR | NR |
| Flavonifractor plautii | NR | NR | NR | ↑ | NR | NR |
| Lachnospiraceae bacterium | NR | NR | NR | ↑ | NR | NR |
| Ruminococcus gnavus | NR | NR | NR | ↑ | NR | NR |
| Faecalibacterium prausnitzii | NR | NR | NR | ↓ | NR | NR |
| Bifidobacterium adolescentis | NR | NR | NR | ↓ | NR | NR |
| Methanobrevibacter smithii | NR | NR | NR | ↓ | NR | NR |
| Bacteroides clarus | NR | NR | NR | ↓ | NR | NR |
| Bacteroides intestinalis | NR | NR | NR | ↓ | NR | NR |
| Bacteroides salyersiae | NR | NR | NR | ↓ | NR | NR |
| Bacteroides stercoris | NR | NR | NR | ↓ | NR | NR |
| Butyrivibrio crossotus | NR | NR | NR | ↓ | NR | NR |
| Clostridium sp | NR | NR | NR | ↓ | NR | NR |
| Coprococcus catus | NR | NR | NR | ↓ | NR | NR |
| Eubacterium hallii | NR | NR | NR | ↓ | NR | NR |
| Eubacterium ramulus | NR | NR | NR | ↓ | NR | NR |
| Odoribacter splanchnicus | NR | NR | NR | ↓ | NR | NR |
| Peptostreptococcaceae noname unclassified | NR | NR | NR | ↓ | NR | NR |
| Prevotella copri | NR | NR | NR | ↓ | NR | NR |
| Ruminococcus callidus | NR | NR | NR | ↓ | NR | NR |
| Ruminococcus champanellensis | NR | NR | NR | ↓ | NR | NR |
| Ruminococcus obeum | NR | NR | NR | ↓ | NR | NR |
| Sutterella wadsworthensis | NR | NR | NR | ↓ | NR | NR |
| **Phylum** |  |  |  |  |  |  |
| Firmicutes | NR | NR | NR | ↑ | NR | NR |
| Bacteroidetes | NR | NR | NR | NR | NR | ↑ |
| Proteobacteria | NR | NR | NR | NR | NR | ↑ |
| Actinobacteria | NR | NR | NR | NR | NR | ↓ |
| Cyanobacteria | NR | NR | NR | NR | ↓ | NR |
| **Class** |  |  |  |  |  |  |
| Tissierellales | NR | ↑ in episodic and chronic vs control | NR | NR | NR | NR |
| Tissierellia | NR | ↑ in episodic and chronic vs control | NR | NR | NR | NR |
| Bacilli | NR | ↑ in episodic vs chronic | NR | NR | NR | NR |
| **family** |  |  |  |  |  |  |
| Peptoniphilaceae | NR | ↑ in episodic vs control  ↑chronic vs control | NR | NR | NR | NR |
| Eubacteriaceaee | NR | ↑ in episodic vs control | NR | NR | NR | NR |
| Selenomonadaceae | NR | ↑ in episodic migraine vs chronic migraine | NR | NR | NR | NR |
| Prevotellaceae | NR | ↑ in episodic migraine vs chronic migraine | NR | NR | NR | NR |
| **Order** |  |  |  |  |  |  |
| Selenomonadales | NR | ↑ in episodic migraine vs chronic migraine | NR | NR | NR | NR |
| Lactobacillales | NR | ↑ in episodic migraine vs chronic migraine | NR | NR | NR | NR |

*Yong et al. explored both episodic and chronic migraine microbiome.
